# Understanding physiological mechanisms of variation in grain filling of maize under high planting density and varying nitrogen applicate rate

**DOI:** 10.3389/fnut.2022.998946

**Published:** 2022-08-24

**Authors:** Hong Ren, Ming Zhao, Baoyuan Zhou, Wenbin Zhou, Kemin Li, Hua Qi, Ying Jiang, Congfeng Li

**Affiliations:** ^1^Institute of Crop Science, Chinese Academy of Agricultural Sciences/Key Laboratory of Crop Physiology and Ecology, Ministry of Agriculture and Rural Affairs, Beijing, China; ^2^College of Agronomy, Shenyang Agricultural University, Shenyang, China

**Keywords:** maize, grain filling, ^13^C-photosynthates, vascular bundle structure, matter transport efficiency

## Abstract

Grain filling is a critical process for achieving a high grain yield in maize (*Zea mays* L.), which can be improved by optimal combination with genotype and nitrogen (N) fertilization. However, the physiological processes of variation in grain filling in hybrids and the underlying mechanisms of carbon (C) and N translocation, particularly under various N fertilizations, remain poorly understood. The field experiment was conducted at Gongzhuling Farm in Jilin, China. In this study, two maize hybrids, i.e., Xianyu 335 (XY335) and Zhengdan958 (ZD958) were grown with N inputs of 0, 150, and 300 kg N ha^–1^ (N0, N150, and N300) in 2015 and 2016. Results showed that the N application significantly optimized grain-filling parameters for both maize hybrids. In particular, there was an increase in the maximum filling rate (*G*_*max*_) and the mean grain-filling rate (*G*_*mean*_) in XY335 by 8.1 and 7.1% compared to ZD958 under the N300 kg ha^–1^ (N300) condition, respectively. Simultaneously, N300 increased the small and big vascular bundles area of phloem, and the number of small vascular bundles in peduncle and cob at the milking stage for XY335. XY335 had higher root bleeding sap (10.4%) and matter transport efficiency (8.4%) of maize under N300 conditions, which greatly enhanced the ^13^C assimilates and higher C and N in grains to facilitate grain filling compared to ZD958. As a result, the grain yield and the sink capacity for XY335 significantly increased by 6.9 and 6.4% compared to ZD958 under N300 conditions. These findings might provide physiological information on appropriate agronomy practices in enhancing the grain-filling rate and grain yield for maize under different N applications, namely the optimization variety and N condition noticeably increased grain filling rate after silking by improving ear vascular structure, matter transport efficiency, and enhancing C and N assimilation translocation to grain, eventually a distinct improvement in the grain sink and the grain yield.

## Introduction

A critical process for achieving high grain yield in maize is grain filling, which is closely associated with kernel number and weight and determined by grain-filling rate (GFR) and period (GFP) (1, 2). The grain filling was affected by nitrogen (N) application and crop genotype, which has been well documented (3, 4). Although increasing plant density improves the grain yield of maize, leaf mutual shading would reduce the pollination rate and photosynthesis, which adversely affect GFR (5) and GFP (6), not only affects grain weight but also kernel number (7, 8). A growing number of studies on N effect on grain filling tend to favor that GFR is more influential than GFP in achieving high grain yield (4, 9). However, the physiological mechanism of N influencing the GFR between two maize hybrids is still unclear, especially under high plant density. Therefore, further research is needed to resolve this question and thus we might gain better insights into the mechanisms of increasing maize grain yield by investigating the grain-filling characteristics between various levels of N inputs.

Three essential factors, assimilate supply, matter transport, and sink capacity, influence grain filling (10–12). Grain filling depends on the grain carbon (C) assimilates obtained from both the C remobilized from reserves of C pools in vegetative organs either pre- or post-anthesis and assimilates currently produced in photosynthetic tissues. Nitrogen has a major role in the initiation of sink size establishment and C (i.e., sucrose) and N assimilates supporting kernel development and growth in interdependent ways during the grain-filling period (13, 14). However, the majority of relevant studies had focused on how N supply alters the final kernel number or N allocation in various parts of plants, while played little attention to how N supply influences C assimilates allocation to kernels during the grain-filling to maturity stages (10, 12, 15). Thus, the determination of C assimilates allocation before and after anthesis would be beneficial to understand the response mechanisms of GFR and GFP between maize hybrids grown with different amounts of N fertilizer.

A robust matter-transport system within a plant is an essential prerequisite for C assimilates allocation from sources to meet the high assimilates requirements of sink establishment, namely kernel development, and growth (16–19). Root bleeding sap is one of the key factors to boost the matter transport system, because its quantity and components reveal the shoot growth potential and root activity (20, 21). Higher matter transport efficiency (MTE) is greatly dependent on the vascular bundle system because this system is the main channel for transporting C and N compounds (14). Both the amount and area of vascular bundles play a crucial role in transporting photosynthates and nutrients (22, 23). Our previous study clearly shows that N fertilizer management increased the number of the small vascular bundle to strongly affect matter transport and crop grain production (9), but played little attention to how crop variety selection and N interaction influence matter transport to grain. Thus, revealing how crop genotype and N application influence the traits of vascular bundles in crops would deepen our understanding of the variability in matter transport and nutrient allocation in crops.

Kernel’s ability to utilize and absorb assimilates was highly dependent on N fertilization and crop genotype. Appropriate high N fertilizer input has been shown to increase dry matter accumulation and distribution to reproductive organs and is associated with better efficiency in the use of C assimilates by kernels (15, 24, 25). However, little information was available about the physiological processes and carbon (C) and N translocation of different maize hybrids in grain filling, particularly under various N fertilizations. Here, we investigated some of the complex relationships involved in maize grain development and yield production as affected by genotype and N supply. Our specific aims were to (1) compare grain-filling attributes and grain yields between two maize hybrids in response to various levels of N supply, (2) reveal underlying mechanisms of C assimilates allocation in the grain-filling process of maize hybrid with high sink capacity and grain yield, (3) and explore how matter transport and vascular bundle characteristics relate to crop grain-filling and differ in response to various N supplies between two maize hybrids.

## Materials and methods

### Experimental site descriptions

A field experiment was conducted at Gongzhuling, Jilin Province (43°31’N, 124°48’E), China during the maize growing season of April–October in 2015 and 2016. The experimental soil was black earth (Hapli-Udic Cambisol) with the following soil properties: pH 6.3, organic matter 26.6 g kg^–1^, total N 1.6 g kg^–1^, available P 62.3 mg kg^–1^, and available K 148.40 mg kg^–1^. These values were obtained from soil sampled from the 0 to 20 cm soil profile before this study. During maize growing seasons, air temperatures above 10°C were summed to calculate the effective cumulative temperature, which was 1631.3°C in 2015 and 1616.0°C in 2016. Cumulative rainfalls were 409.6 mm in 2015 and 643.7 mm in 2016.

### Experimental design and crop management

In a split-plot design, two maize hybrids were established as the main plots and three N levels were established as subplots, totaling 18 plots with three replications. Each plot was 45 m^2^ in area (7.5 m length × 6 m width). Two widely grown high-yielding spring maize varieties, Xianyu 335 (XY335; It was bred by the American Pioneer Company. The parental inbred lines are PH4CV and PH6WC. PH6WC and PH4CV come from the SS and NSS heterotic groups of M America, respectively.) and Zhengdan 958 (ZD958; It was bred by the Henan Academy of Agricultural Sciences. The parental inbred lines are Zheng58 and Chang7-2, which came from the PA and SPT heterotic groups in China, respectively.), were planted in the main plots. Three N fertilizer (urea) levels, 0 kg ha^–1^ (N0), 150 kg ha^–1^ (N150), and 300 kg ha^–1^ (N300), were individually applied in the subplots. The N was applied before the sowing, jointing, and silking stages of maize at a ratio of 5:3:2 for the three applications. Both phosphorus [Ca_3_(PO_4_)_2_] and potassium (KCl) fertilizers were applied at 100 kg ha^–1^ before sowing in 2015 and 2016. Maize was planted in rows with a 60 cm row spacing and 90,000 pl ha^–1^ density on April 29th and April 30th and manually harvested on October 1st and September 30th in 2015 and 2016, respectively. Pests, weeds, and diseases were well-controlled and no irrigation was applied throughout the two growing seasons.

### Data collection

#### Sampling and grain-filling parameters

From the beginning of maize pollination, 50 plants that visually appeared uniform in growth were marked in each plot to record the date of ear pollination. Three ears among the marked pollinated plants were collected every 7–15 days for a total of five time points in 2015 and six time points in 2016 (19). We then collected 100 kernels from the middle part of each ear and initially dried them in an oven at 105°C for 40 min before drying to a constant weight at 80°C. Then we determined the 100-kernel weight as a measure of the grain-filling process by fitting a logistic equation (Eq. 1) according to Wei et al. (4).


(1) $$W=A/\left(1+B\,E\,X\,P^{-C\,t}\right)$$

In the above equation, W is the 100-kernel weight (g) and t is the number of days after pollination. The estimated parameters A, B, and C represent final mass, the coefficient at the initial stage, and growth rate, respectively. A second equation (Eq. 2), derived by taking the first derivative of Eq. 1 (4), was used to estimate effective grain-filling duration and kernel growth rate:


(2) $$D\,w/d\,t=A\times B\times C\times E\,X\,P^{-C\,t}/{\left(1+B\times E\,X\,P^{-C\,t}\right)}^{2}$$

The following equations describe the determination of additional grain-filling parameters of maize. Kernel weight at the maximum grain-filling rate was determined by(*Wmax*) = *A*/2. The maximum grain filling rate equation is (*Gmax*) = (*C*×*Wmax*)×[1−(*Wmax*/*A*)]. The mean grain filling rate equation is (*Gmean*) = (*A*/2)×(*C*/6). The active grain-filling period was determined by (*P*) = 6/*C*.

#### Root activity (TTC reducing capacity) and malondialdehyde content

At the milking stage, 0–60 cm of soil root was selected, then it was divided into 0–15,15–30, and 30–60 cm of three layers to measure the root activity and malondialdehyde (MDA) content in 2015 and 2016. Root activity (TTC reducing capacity) was measured according to the method of Duncan and Widholm (26).

Malondialdehyde (MDA) content was measured as follows: 0.3 g root was selected in each sample, 2 ml 10% TCA solution was added, and then it was finely ground. Then, it was poured into a centrifugal tube, 6 mL TCA was added to wash, put the homogenate into a centrifuge tube (4,000 r/min for 10 min), and the supernatant was collected. Take 2 mL supernatant, add into 2 mL 0.6% TBA solution, mix and plug in the tube, put into a seal with plastic wrap, kept the mixture at 100°C for another 30 min. Taking supernatant to obtain OD values at 532 and 450 nm. The CK is a TCA solution. Finally, calculate the MDA content (μmol/g) by C(μ*molL*^−1^) = 6.45×*A*532−0.56×*A*450.

### ^13^C-photosynthate distribution and C/N ratio between plant organs

We used the ^13^C isotope as a tracer in a labeling experiment to evaluate the effect of maize hybrids and N fertilizer levels on the ^13^C-photosynthate distribution among plant organs in 2016. Six plants of robust and uniform growth were selected in each plot for ^13^C-labeling at the third day after silking. Mylar plastic bags (length 1 m, width 15 cm, and thickness 0.1 mm) were used to encase the ear leaf. Then, 50 ml of ^13^CO_2_ was injected into the bags. After the enclosed leaves were allowed to continue photosynthesizing for 60 min, the bags were removed from the ear leaf in each plot.

Labeled plants from each plot were harvested at two time points. The first set of three plants was sampled 24 h after the ^13^C-labeling of leaves. The remaining three ^13^CO_2_-labeled plants were harvested when they reached the physiological maturity stage (R6). All plant samples were divided into ear leaves, other leaves, stem, sheath, cob, ear bracts, and grain. All plant materials were heated at 105°C for 1.5 h and then dried to constant weights at 80°C before milling into fine powders. Using 5 mg of each powdered sample, we determined isotopic abundance using an Isoprime 100 instrument (Isoprime100, Cheadle, United Kingdom). Significance analysis was performed on the same growth stage between treatments at a 5% level. For C and N content determination in 2016, all leaf fractions from each plant were mixed together as a single leaf sample and then analyzed along with the remaining stem and grain samples according to the method mentioned in a previous study (27).

#### Vascular bundles number and area and matter transport efficiency

At the maize milking stage of the 2016 growing season, the plant fractions of the basal-stem, peduncle internode, and cob internode were obtained from five plants per in each plot according to Piao et al. (28). The plant samples were fixed using the Kano fixative solution (*V*_*acetic acid*_/*V*_*alcohol*_ = 1:3) and were stored in 70% ethanol solution before obtaining images of vascular bundle structure. Images were captured using a Zeiss Axio Scope with a 5 × /0.3 numerical aperture and a 10 × /0.3 NA Axio HRc camera (Carl Zeiss Inc., Ontario, CA, United States). Then, we analyzed images using the ZEN analysis system (Axio Lab A1, Zeiss, Germany) to obtain relevant data regarding the area occupied by large and small vascular bundles and xylem and phloem per vascular bundle. The average values from 18 adjacent vascular bundles were recorded for each treatment. Significant analysis was performed on the same positions between treatments at a 5% level.

Root bleeding sap was collected from at the basal internode of the stem. The protocol for the collection of sap was according to the method described in previous studies by Piao et al. (28). Then, the total areas of big/small vascular bundles were calculated according to Eq. 3. The matter transport efficiency (MTE, mg mm^–2^ h^–1^) was calculated using Eq. 4 (28).


(3) Total⁢area⁢of⁢big/small⁢vascular⁢bundle=signal⁢area⁢of⁢big/small⁢vascular⁢bundle×total⁢number⁢of⁢big/small⁢vascular⁢bundle

(4) M⁢T⁢E=R⁢B⁢S/V⁢A⁢B

Here, *RBS* refers to the rate of root bleeding-sap collected from 17:00 to 05:00 of the next day (mg h^–1^), and *VBA* refers to the vascular bundle area in the basal stem internode (mm^2^).

#### Count of florets, grain yield, kernel number per spike, and 1,000-kernel weight

On the 10th day after maize pollination in 2015 and 2016, the husk leaves were removed from 10 ears selected from each plot after the total number of florets per ear was recorded. The number of pollinated florets included two counts by simply shaking: Both falling and withered silks in ovary silk junction were counted as the number of fertilized florets. The number of fresh silk that was not fell off was recorded as the number of unpollinated florets (29).

At the maturity stage of maize, four rows of maize in each plot were harvested to determine grain yield (grain yields were standardized to 14% moisture), kernel number per plant, and 1,000-kernel weight. Sink capacity was determined by Eq. 5 as described by Yoshinaga et al. (30).


(5) Sink⁢capacity=KNP×KW×plant⁢number⁢per⁢unit⁢area

Here, *KNP* refers to the kernel number per ear^–1^, *KW* is kernel weight and *the plant numbers per unit area* were obtained from a 1 m^2^ area in each plot.

## Results

### Grain components, grain yield, and sink capacity

There were significant effects from the factors of Year (Y), Nitrogen (N), and Genotype (G) on kernel number per ear^–1^ (KNP), 1,000-kernel weight (TKW), grain yield (Y effect on grain yield not included), sink capacity, number of pollinated florets (NFP), and the total number of florets (TNF) (Table 1). For both varieties, increasing the levels of N applied to soils significantly increased KNP by an average of 10.8% and TKW by 9.2% between N150 and N300. Increasing the levels of N applied grain yield increases by an average of 22.7% and sink capacity by an average of 20.6% between N150 and N300. Interestingly, averagely a lower TKW (6.4%) but a higher NPF (7.3%) and KNP (12.4%) were observed for XY335 compared to those for ZD958, which contributed to 7.5% (2015) and 6.3% (2016) increases in KWP of XY335 from that of ZD958 under the N300 treatment. Conversely, under the N0 and N150 treatments, KWP values for ZD958 compared to those for XY335 appeared greater but without significant difference (Table 1).

**TABLE 1:** Effect of nitrogen fertilization level on grain yield component and sink capacity between two maize hybrids in 2015 and 2016.

Year	Genotype	N level	KNP	TKW	GY	SC	NPF	TNF
			(ear^–1^)	(g)	(kg ha^–1^)	(g m^–2^)	(No.)	(No.)
2015	XY335	N0	335 ± 13.5d	233 ± 3.3e	5445 ± 31.8f	626 ± 33.1f	478 ± 1.1e	396 ± 11.7d
		N150	410 ± 4.4c	271 ± 4.5c	8992 ± 48.0d	940 ± 6.0d	698 ± 4.7c	500 ± 10.8c
		N300	505 ± 13.0a	286 ± 3.6b	12313 ± 28.8a	1258 ± 14.4a	760 ± 7.2a	601 ± 3.5a
	ZD958	N0	336 ± 4.8d	253 ± 1.3d	6073 ± 33.3e	681 ± 7.7e	472 ± 4.4e	389 ± 7.7d
		N150	415 ± 12.6bc	283 ± 1.3b	9568 ± 82.9c	999 ± 10.3c	662 ± 8.8d	499 ± 12.2c
		N300	432 ± 16.0b	315 ± 4.5a	11387 ± 19.0b	1184 ± 20.3b	717 ± 8.3b	565 ± 11.0b
2016	XY335	N0	321 ± 16.0d	229 ± 0.5f	4778 ± 52.5f	588 ± 28.1f	538 ± 4.0d	384 ± 4.6d
		N150	460 ± 12.0bc	280 ± 1.9d	8984 ± 23.4d	1068 ± 20.1d	727 ± 9.0b	539 ± 4.2c
		N300	535 ± 10.1a	328 ± 2.3b	12627 ± 35.6a	1438 ± 12.2a	771 ± 5.9a	633 ± 17.0a
	ZD958	N0	312 ± 2.0d	273 ± 1.6e	5485 ± 50.4e	681 ± 6.8e	524 ± 5.3d	360 ± 20.0d
		N150	449 ± 1.2c	317 ± 2.3c	9602 ± 69.4c	1128 ± 16.7c	675 ± 14.5c	518 ± 28.0c
		N300	480 ± 16.0b	340 ± 3.7a	11836 ± 39.3b	1339 ± 25.3b	702 ± 19.0bc	590 ± 19.7b
ANOVA	Year (Y)		***	***	NS	***	***	*
	Nitrogen (N)		***	***	***	***	***	***
	Genotype (G)		***	***	*	*	***	***
	N × G		***	NS	***	***	***	NS
	Y × N × G		***	NS	***	NS	***	**

KNP, Kernel number per ear^–1^; TKW, 1,000-kernel weight; GY, Grain yield; SC, Sink capacity; NPF, Number of pollinated florets; TNF, Total number of florets. N0, N150, and N300 indicate 0, 150, and 300 kg ha^–1^ N applied, respectively.

Different letters indicate significant differences between treatments at a 5% level.

*, **, and *** indicate different significance at 5, 1, and 0.1% level, respectively.

NS, no significance.

### Grain filling characteristics

**FIGURE 1:**
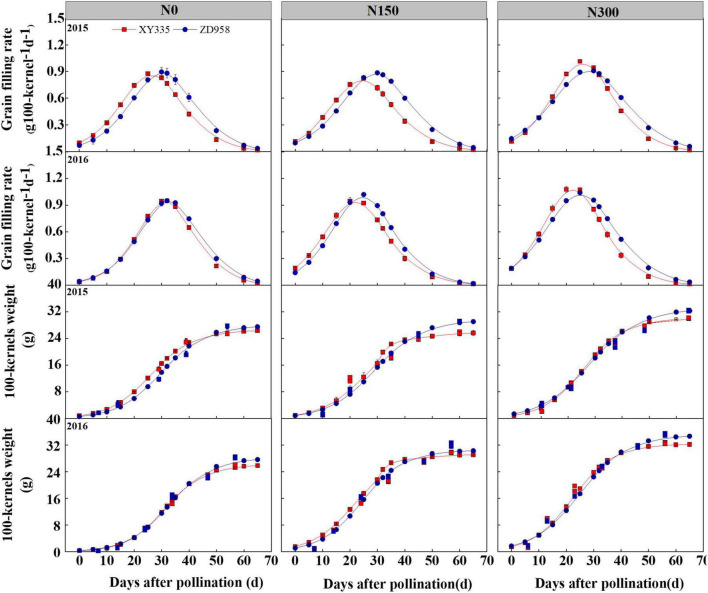
Dynamics of 100-kernel weight and grain filling rate of two maize hybrids after pollination under various N levels applied in 2015 and 2016. N0, N150, and N300 indicate 0, 150, and 300 kg N ha^– 1^ applied, respectively. The values shown are the mean ± SE (*n* = 3).

After maize pollination, the changes in 100-kernel weight for both varieties appeared in three stages of increasing change from gradual to rapid to slight increases over time (Figure 1). Initially, there was no obvious difference between varieties, when the weights were gradually increased. Then the 100-kernel weight of XY335 rose higher than that of ZD958 when both weights were rapidly increasing in the N0 and N150 treatments in 2015. Accordingly, for grain-filling rate, XY335 presented higher and lower values at the time periods of 14–32 and 35–60 days after pollination compared to that of ZD958 in 2015, respectively. Similar trends were observed in the 2016 growth season as well but with slightly higher and lower differences between varieties. Generally, XY335 achieved *G*_*max*_ sooner than ZD958 achieved it in each study year (Figure 1).

In general, increasing N levels promoted *W*_*max*_, *G*_*max*_, and *G*_*mean*_ for both maize varieties, particularly in N300. Higher *G*_*max*_ and *G*_*mean*_ values were observed for ZD958 in comparison to those of XY335 in N0 and N150 conditions, whereas the *G*_*max*_ and *G*_*mean*_ values obtained from ZD958 were on average 8.1 and 7.1% lower than the respective values from XY335 in the N300 conditions. Notably, XY335 had shorter GFPs on averages of 5.3, 2.7, and 15.4% than those of ZD958 in the N0, N150, and N300 levels, respectively (Table 2).

**TABLE 2:** Effect of nitrogen fertilization level on grain filling parameters between two maize hybrids in 2015 and 2016.

Year	Genotype		N level	*W*_*max*_ (mg kernel^–1^ d^–1^)	*T*_*max*_ (d)	*G*_*max*_ (mg kernel^–1^ d^–1^)	GFP (d)	*G*_*mean*_ (mg kernel^–1^ d^–1^)
2015	XY335		N0	132.2 ± 1.52d	26.3 ± 0.33c	8.8 ± 0.04d	44.9 ± 0.73d	0.29 ± 0.00d
			N150	128.8 ± 4.20d	25.5 ± 2.04c	8.4 ± 0.38d	46.2 ± 0.90c	0.28 ± 0.01d
			N300	149.9 ± 0.41b	25.9 ± 0.30c	10.2 ± 0.12a	44.2 ± 0.63d	0.34 ± 0.00a
	ZD958		N0	138.9 ± 3.27c	30.1 ± 0.61a	9.0 ± 0.26bc	46.5 ± 0.36c	0.30 ± 0.01c
			N150	147.0 ± 0.38b	29.3 ± 0.22ab	8.9 ± 0.06c	49.8 ± 0.44b	0.30 ± 0.01c
			N300	163.8 ± 0.71a	28.1 ± 0.39b	9.2 ± 0.02b	53.6 ± 0.37a	0.31 ± 0.01b
2016	XY335		N0	129.9 ± 2.16f	31.3 ± 0.16b	9.5 ± 0.09c	40.9 ± 0.96c	0.32 ± 0.01c
			N150	145.8 ± 1.24d	22.0 ± 0.39d	9.6 ± 0.37c	45.5 ± 2.10b	0.32 ± 0.01c
			N300	161.4 ± 1.68b	22.4 ± 0.50d	11.1 ± 0.24a	43.7 ± 1.15b	0.37 ± 0.01a
	ZD958		N0	139.7 ± 0.68e	32.7 ± 0.43a	9.5 ± 0.23c	44.1 ± 1.27b	0.32 ± 0.01c
			N150	151.9 ± 1.63c	24.6 ± 0.05c	10.2 ± 0.08b	44.7 ± 0.51b	0.34 ± 0.00b
			N300	174.7 ± 0.70a	25.1 ± 0.12c	10.4 ± 0.01b	50.4 ± 0.27a	0.35 ± 0.00b
ANOVA	Year (Y)			***	***	***	***	***
	Nitrogen (N)			***	***	***	***	***
	Genotype (G)			***	***	NS	***	NS
	N × G			*	NS	***	***	***
	Y × N × G			***	NS	NS	**	NS

W_max_, kernel weight increment achieving maximum grain-filling rate; T_max_, the days reaching the maximum grain-filling rate; G_max_, maximum filling rate; GFP, active filling phase; G_mean_, mean grain-filling rate. N0, N150, and N300 indicate 0, 150, and 300 kg ha^–1^ N applied, respectively.

Different letters indicate significant differences between treatments at a 5% level.

*, **, and *** indicate different significance at 5, 1, and 0.1% level, respectively.

NS, no significance.

### C and N contents and C/N ratio in maize organs

**FIGURE 2:**
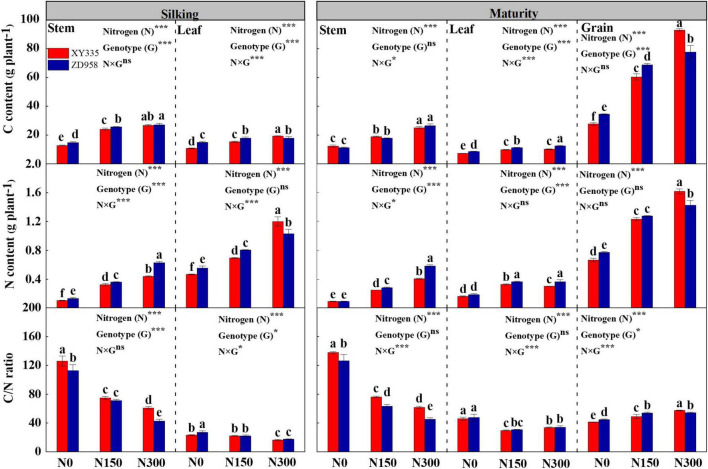
N content, C content, and C/N ratio in stem, leaf, and grain of two maize hybrids at silking and maturity stages under various N levels applied in 2016. N0, N150, and N300 indicate 0, 150, and 300 kg N ha^– 1^ applied, respectively. Different letters indicate significant differences at a 5% level. The values shown are the mean ± SE (*n* = 3). The values shown are the mean ± SE (*n* = 3). *p < 0.05, **p < 0.01, ***p < 0.001, ns, no significance.

In maize stems, N application (N150 and N300) significantly increased C and N contents at both the silking and maturity stages compared to those of N0 (Figure 2). At both growth stages, the C/N ratio in stems decreased gradually with the increase in amounts of applied N. Additionally, significantly greater N contents were measured in ZD958 stems than in XY335 stems grown under N-treated conditions, which resulted in higher C/N ratios in the stems of XY335 than in ZD958. Most notably, significantly lower C contents were recorded from leaves of XY335 than from leaves of ZD958 at the silking stage in the N0 and N150, while in the N300 treatment they showed the opposite observations in the N0 and N150 treatments. The leaf C and N contents were observed significantly lower in XY335 than that of ZD958 at the maturity stage. As a result, the grains of XY335 were, respectively, 19.8–12.3% and 13.7–3.5% lower in C and N contents compared with those in the grains of ZD958 in the N0 and N150, while higher 16.5% for C and 11.8% for N contents than those of ZD958 in N300 treatment. XY335 performed 5.4% higher C/N ratios than ZD958 under N300 conditions (Figure 2), probably suggesting that differences between the two cultivars in matter translocation from source to sink occurred from the silking to maturity stages of maize.

### Root activity and malondialdehyde contents

**FIGURE 3:**
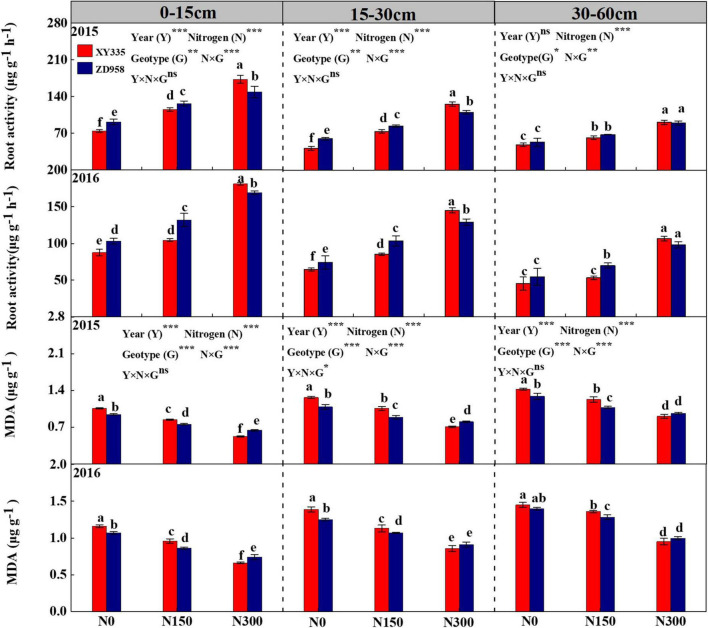
Root activity and malondialdehyde (MDA) content of two maize hybrids at the milking stage under various N levels were applied in 2015 and 2016. N0, N150, and N300 indicate 0, 150, and 300 kg N ha^– 1^ applied, respectively. The values shown are the mean ± SE (*n* = 3). *p < 0.05, **p < 0.01, ***p < 0.001, ns, no significance.

Root activity (Year effected on root activity in 30–60 cm not included) and malondialdehyde (MDA) contents were significantly affected by the factors Year (Y), Nitrogen (N), genotype (G), and N × G. Root activity levels gradually raised with the increase of N inputs, while MDA was reduced with increased N rate. As soil depth increased, root activity was reduced, and MDA was observed as an enhanced trend in each N level condition (Figure 3). During 2 years, root activity within the 0-60 cm soil layer samples from XY335 was significantly lower than that of ZD958 in the N0 (17.4%) and N150 (15.4%) treatments. Conversely, greater root activity was observed in XY335 higher than those in ZD958 by averages of 8.9% at the N300 levels. MDA contents in root were higher for XY335 than those for ZD958 by averages of 9.3 and 10.0% in N0 and N150 treatment, while lower 9.7% in XY335 than that of ZD958 at the N300 N level (Figure 3).

### Distribution of ^13^C-photosynthates in tissues at maize silking and maturity stages

The distribution of ^13^C-photosynthates in each tissue of two maize cultivars was significantly affected by the levels of applied N; however, significant effects on two varieties were only observed from the ^13^C-photosynthates distributions in the sheath, grain, and cob tissues (Table 3). At the silking stage, similar distribution patterns of ^13^C-photosynthates in tissues were obtained in the same N-treated plants of both cultivars. Moreover, significantly higher amounts of ^13^C-photosynthates were distributed in the stems and husk leaves of crops from the N150 and N300 treatments relative to those from the N0 treatment. Accordingly, those labeled-^13^C captured by other leaves and sheaths were lower in N input treatments than those in treatment without N fertilizer. These results indicated that N input accelerated ^13^C-photosynthate allocation to stem and husk leaves at the silking stage.

**TABLE 3:** Effect of nitrogen fertilization level on the distribution of ^13^C-photosynthates among tissues of two maize hybrids at silking and maturity stages in 2015 and 2016.

Year	Growth stages	Genotype	N level	^13^C-photosynthates distribution in different tissues of maize (%)						
				Stem	Ear leaf	Other leaf	Sheath	Husk leaf	Grain	Cob
2015	Silking	XY335	N0	37.0 ± 1.05c	2.7 ± 0.20c	35.1 ± 1.39ab	20.6 ± 0.54a	4.5 ± 0.40bc	–	–
			N150	40.5 ± 0.42b	2.9 ± 0.16c	33.5 ± 1.01b	17.4 ± 1.22c	5.6 ± 0.20a	–	–
			N300	43.3 ± 0.48a	3.5 ± 0.21ab	31.4 ± 1.10c	16.5 ± 0.46cd	5.3 ± 0.51ab	–	–
		ZD958	N0	36.6 ± 1.33c	3.1 ± 0.27bc	36.4 ± 0.90a	19.3 ± 0.37b	4.6 ± 0.22bc	–	–
			N150	41.5 ± 1.21b	3.6 ± 0.16a	33.7 ± 0.01b	16.9 ± 0.66cd	4.4 ± 0.38c	–	–
			N300	44.4 ± 0.51a	3.3 ± 0.14bc	30.8 ± 0.47c	15.8 ± 0.76d	5.7 ± 0.42a	–	–
	Maturity	XY335	N0	17.9 ± 0.07c	1.3 ± 0.13a	14.0 ± 0.48b	7.1 ± 0.67b	6.4 ± 0.38a	45.5 ± 0.39d	7.5 ± 0.60a
			N150	18.7 ± 0.35b	0.9 ± 0.16ab	10.5 ± 0.28c	6.6 ± 0.47bc	6.5 ± 0.17a	51.9 ± 0.42c	5.1 ± 0.28bc
			N300	18.7 ± 0.59b	0.9 ± 0.02ab	8.6 ± 0.21d	6.0 ± 0.43c	6.6 ± 0.08a	54.9 ± 0.37a	4.6 ± 0.22c
		ZD958	N0	16.3 ± 0.19d	1.2 ± 0.10a	15.3 ± 0.98a	9.7 ± 0.35a	5.5 ± 0.24ab	45.0 ± 0.52d	6.9 ± 0.97a
			N150	18.5 ± 0.34b	0.9 ± 0.12ab	12.4 ± 0.58c	6.1 ± 0.61c	5.5 ± 0.46ab	50.8 ± 0.49c	5.8 ± 0.23b
			N300	19.5 ± 0.47a	0.8 ± 0.02b	10.4 ± 0.52c	6.0 ± 0.53c	4.9 ± 0.25b	53.3 ± 1.18b	5.2 ± 0.14bc
2016	Silking	XY335	N0	36.1 ± 1.47c	3.8 ± 0.20a	33.3 ± 1.85a	23.8 ± 0.53a	3.0 ± 0.16c	–	–
			N150	43.2 ± 0.69b	3.2 ± 0.13c	29.9 ± 0.68bc	18.5 ± 0.69bc	5.2 ± 0.52a	–	–
			N300	46.2 ± 1.96a	3.5 ± 0.16ab	28.0 ± 2.63bc	16.9 ± 1.11c	5.4 ± 0.46a	–	–
		ZD958	N0	35.7 ± 0.97c	3.2 ± 0.17c	34.2 ± 2.27a	22.6 ± 1.15a	4.3 ± 0.69b	–	–
			N150	42.6 ± 0.94b	3.4 ± 0.19bc	29.0 ± 0.73bc	19.8 ± 1.02b	5.2 ± 0.15a	–	–
			N300	47.3 ± 1.58a	3.3 ± 0.11bc	26.2 ± 1.64c	18.1 ± 0.53c	5.1 ± 0.31a	–	–
	Maturity	XY335	N0	18.6 ± 0.78b	1.9 ± 0.11ab	11.9 ± 0.29b	11.2 ± 0.33a	4.5 ± 0.53a	44.2 ± 0.44d	7.7 ± 0.07a
			N150	18.1 ± 0.54bc	1.4 ± 0.02b	9.6 ± 0.19c	7.3 ± 0.24cd	4.6 ± 0.32a	52.4 ± 0.65b	6.6 ± 0.12b
			N300	19.1 ± 0.70b	1.6 ± 0.02b	6.9 ± 0.23d	6.1 ± 0.22e	3.9 ± 0.05b	56.6 ± 0.68a	5.9 ± 0.37bc
		ZD958	N0	17.1 ± 0.80c	2.0 ± 0.57a	13.2 ± 0.18a	11.9 ± 0.63a	3.4 ± 0.44b	46.0 ± 0.99c	6.4 ± 0.77b
			N150	18.2 ± 0.74bc	1.7 ± 0.06ab	11.8 ± 0.43b	7.7 ± 0.85bc	3.7 ± 0.32b	51.0 ± 0.92b	5.9 ± 0.23bc
			N300	20.3 ± 0.18a	1.5 ± 0.11b	9.3 ± 0.28c	6.5 ± 0.52de	2.1 ± 0.08c	55.1 ± 0.54a	5.2 ± 0.22c
ANOVA	Year (Y)			NS	NS	NS	NS	***	***	**
	Nitrogen (N)			*	*	***	***	***	***	*
	Genotype (G)			NS	NS	NS	*	NS	**	***
	N × G			NS	NS	NS	NS	NS	NS	*
	Y × N × G			NS	NS	NS	NS	NS	NS	**

N0, N150, and N300 indicate 0, 150, and 300 kg ha^–1^ N applied, respectively.

Different letters in the same column indicate significant differences between treatments at a 5% level for each growth stage.

*, **, and ** indicate different significance at 5, 1, and 0.1% level, respectively.

NS, no significance; –, no data for use.

At maturity, the ^13^C photosynthetic products in source tissues were transferred to grains in large quantities, and grains as sinks then became the organs containing the most ^13^C photosynthetic products. XY335 exhibited relatively higher ^13^C-photosynthate allocation ratios in grain, cob, and husk leaves than ZD958 exhibited in the corresponding tissues. As expected, XY335 exhibited relatively lower ^13^C-photosynthate allocation in other tissues than ZD958 exhibited in tissues, particularly in leaves. The ratio was lower by 9.8, 18.6, and 25.8% in XY335 than that in ZD958 in the respective N0, N100, and N300 treatments. ^13^C-photosynthate allocation was significantly reduced in other leaves, sheath, and cob, while it was greater in grains for both maize varieties due to the increased N inputs. Under N300 conditions the ratio of ^13^C-photosynthate allocation was increased by 14.7% for XY335, and 12.0% for ZD958 from that in N0 and N150 treatment (Table 3).

### Traits of vascular bundles in internodes of maize

Overall, the area, number, and density of vascular bundles, regardless of whether the sizes of bundles were categorized as small or large, were significantly affected by N fertilization. Furthermore, the area and number of small vascular bundles and vascular bundle density were significantly influenced by the factor of genotype (Table 4). Increased N fertilization levels vastly raised vascular bundle area, and increasing trends were observed in the xylem and phloem of basal-stem, peduncle, and cob samples for both genotypes at the milking stage. In N0 and N150 treatments, XY335 had a relatively lower total area and total phloem area of small vascular bundles than ZD958. However, in the N300 treatment, XY335 had a relatively higher total area of small vascular bundles than ZD958, particularly in peduncles because of the significantly larger area of phloem. Similar results were also observed for the area of small vascular bundles due to the larger area of either the xylem or phloem in basal stems and cobs of maize in the N300 treatment.

**TABLE 4:** Effect of nitrogen fertilization level on vascular bundle traits of two maize hybrids at milking stage in 2016.

Position	Genotype	N level	Area of big vascular bundle (mm^2^)			Area of small vascular bundle (mm^2^)			Number of vascular bundle		Vascular bundle
			Xylem	Phloem	Total	Xylem	Phloem	Total	Big	Small	density (mm^–2^)
Basal-stem	XY335	N0	3.76 ± 0.16d	0.75 ± 0.07e	8.34 ± 0.36d	2.30 ± 0.31cd	1.55 ± 0.02f	17.42 ± 1.27c	115.4 ± 5.4d	574.8 ± 19.3c	2.85 ± 0.22a
		N150	6.27 ± 0.67b	1.61 ± 0.23c	13.16 ± 1.43b	3.81 ± 0.71b	2.05 ± 0.01d	19.18 ± 0.99b	187.2 ± 10.3b	666.0 ± 23.5bc	3.10 ± 0.19a
		N300	10.19 ± 0.55a	3.65 ± 0.23a	27.99 ± 1.61a	4.70 ± 0.47a	3.39 ± 0.06a	28.55 ± 0.85a	289.7 ± 9.3a	758.9 ± 16.2a	2.87 ± 0.08a
	ZD958	N0	4.95 ± 0.14c	1.09 ± 0.06d	10.56 ± 0.33c	2.03 ± 0.08d	1.66 ± 0.02e	13.86 ± 0.82d	141.5 ± 1.3c	450.4 ± 17.0d	2.46 ± 0.15b
		N150	6.20 ± 0.39b	1.43 ± 0.08c	14.30 ± 0.75b	3.00 ± 0.18c	2.48 ± 0.02c	16.53 ± 0.72c	184.7 ± 10.4b	562.5 ± 10.2c	2.47 ± 0.16b
		N300	10.27 ± 0.46a	3.30 ± 0.16b	27.73 ± 1.37a	4.26 ± 0.37b	2.96 ± 0.17b	27.52 ± 0.82a	280.9 ± 12.9a	756.4 ± 17.1a	2.49 ± 0.08b
Peduncle	XY335	N0	2.24 ± 0.38c	1.16 ± 0.19c	6.18 ± 0.24c	1.31 ± 0.21d	0.30 ± 0.02f	4.48 ± 0.41d	111.9 ± 4.4c	230.1 ± 12.2d	3.86 ± 0.09b
		N150	3.84 ± 0.88b	2.07 ± 0.27bc	12.36 ± 1.05b	2.20 ± 0.25c	0.61 ± 0.02d	9.00 ± 0.12c	144.9 ± 5.6b	350.4 ± 7.1c	4.82 ± 0.26a
		N300	9.23 ± 1.24a	4.91 ± 0.60a	22.92 ± 1.63a	3.71 ± 0.11a	1.45 ± 0.03a	14.54 ± 0.29a	224.6 ± 4.1a	456.7 ± 8.9a	4.72 ± 0.24a
	ZD958	N0	2.30 ± 0.08bc	1.24 ± 0.13c	6.74 ± 0.74c	1.28 ± 0.20d	0.40 ± 0.04e	5.23 ± 0.72d	116.5 ± 2.8c	224.7 ± 10.7d	2.77 ± 0.29d
		N150	3.32 ± 0.26bc	1.77 ± 0.12c	11.57 ± 0.63b	2.15 ± 0.01c	0.73 ± 0.01c	10.05 ± 0.42c	131.1 ± 8.2b	343.1 ± 1.0c	3.23 ± 0.08bc
		N300	8.72 ± 1.26a	4.74 ± 0.55a	22.11 ± 1.43a	3.11 ± 0.05b	1.27 ± 0.04b	12.26 ± 0.37b	219.3 ± 7.3a	408.5 ± 2.5b	3.51 ± 0.14bc
Cob	XY335	N0	0.97 ± 0.22c	0.49 ± 0.08c	5.20 ± 0.82d	0.65 ± 0.06d	0.12 ± 0.01e	1.77 ± 0.20e	57.5 ± 7.7c	91.0 ± 7.4d	0.64 ± 0.01bc
		N150	1.79 ± 0.09b	1.39 ± 0.10b	6.32 ± 0.33bc	1.47 ± 0.13c	0.26 ± 0.01d	3.74 ± 0.09d	63.8 ± 3.8bc	145.6 ± 4.8b	0.71 ± 0.02a
		N300	3.02 ± 0.16a	1.87 ± 0.10a	8.75 ± 0.11a	2.74 ± 0.08a	0.49 ± 0.03a	7.03 ± 0.32a	80.8 ± 1.0a	158.6 ± 7.7a	0.75 ± 0.02a
	ZD958	N0	1.09 ± 0.20c	0.47 ± 0.08c	5.42 ± 0.95cd	0.69 ± 0.04d	0.16 ± 0.01e	2.12 ± 0.08e	58.0 ± 5.0c	93.9 ± 5.9d	0.58 ± 0.03c
		N150	1.99 ± 0.13b	1.46 ± 0.07b	7.10 ± 0.31b	1.37 ± 0.26c	0.32 ± 0.02c	4.33 ± 0.24c	72.2 ± 3.2ab	138.4 ± 3.0bc	0.62 ± 0.01bc
		N300	3.14 ± 0.17a	1.84 ± 0.07a	8.92 ± 0.35a	2.11 ± 0.24b	0.45 ± 0.01b	6.50 ± 0.55b	82.6 ± 1.3a	146.2 ± 7.8b	0.61 ± 0.03bc
ANOVA	Nitrogen (N)		***	***	***	***	***	***	***	***	***
	Genotype (G)		NS	NS	NS	***	***	***	NS	**	***
	N × G		NS	NS	NS	NS	***	*	NS	NS	NS

N0, N150, and N300 indicate 0, 150, and 300 kg ha^–1^ N applied, respectively. AVE indicates the average value from the N treatment.

Different letters in the same column indicate significant differences between treatments at a 5% level for each tissue position.

*, **, and *** indicate different significance at 5, 1, and 0.1% level, respectively.

NS, no significance.

**APPENDIX FIGURE 1:**
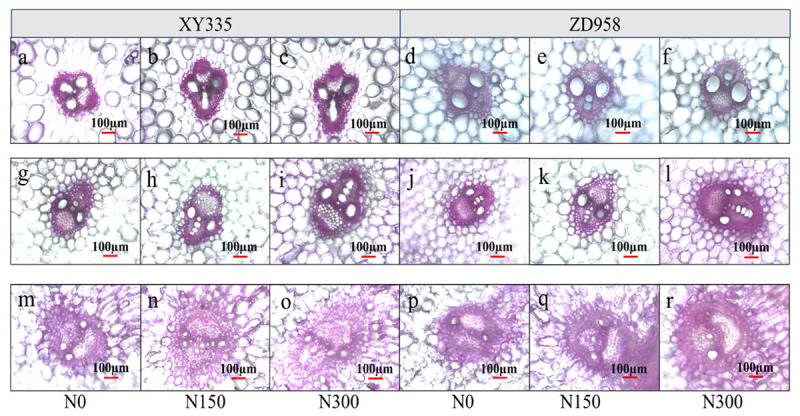
The micrograph of big vascular bundles structure at the basal stem internode of XY335 **(a–c)**, ZD958 **(d–f)**, the peduncle internode of XY335 **(g–i)** and ZD958 **(j–l)**, and the cob internode of XY335 **(m–o)** and ZD958 **(p–r)**. N0, N150, and N300 indicate N applied at 0, 150, and 300 kg ha^– 1^ levels, respectively.

**APPENDIX FIGURE 2:**
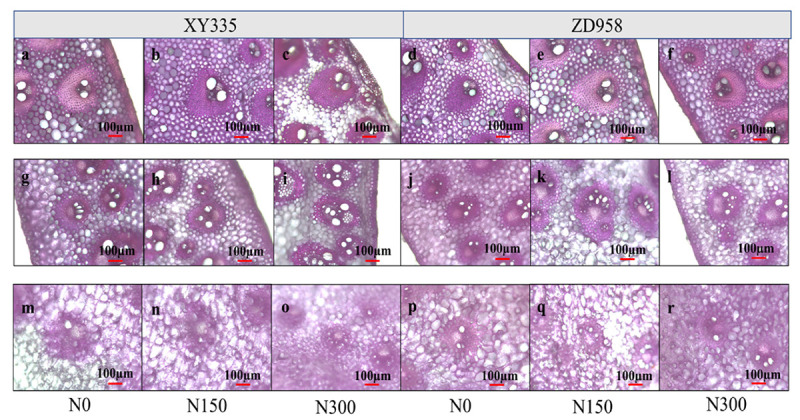
The micrograph of small vascular bundles structure at the basal stem internode of XY335 **(a–c)**, ZD958 **(d–f)**, the peduncle internode of XY335 **(g–i)**, and ZD958 **(j–l)**, and the cob internode of XY335 **(m–o)** and ZD958 **(p–r)**. N0, N150, and N300 indicate N applied at 0, 150, and 300 kg ha^– 1^ levels, respectively.

Similar to the results of the vascular bundle area, the numbers of both large and small vascular bundles were significantly increased by N inputs to both maize varieties. In addition, the number of small vascular bundles was respectively greater on average by 10.6 and 7.8% in the peduncle and cob tissues of XY335 than of ZD958 in the N300 treatment (Table 4). Combining the results of area and number of vascular bundles, XY335 clearly produced a higher vascular bundle density than ZD958 in each tissue of maize no matter what level of N was supplied. The micrographs of vascular bundles of different internodes are presented in Appendix Figures 1, 2.

### Root bleeding-sap and matter transport efficiency

Maize crops grown under the N150 and N300 conditions for both varieties produced approximately 1.5–2.8-fold more root bleeding-sap than crops grown under the N0 treatment produced at the silking stage (Table 5). XY335 had a lower cross-sectional area than that of ZD958 both under N150 and N300 conditions, while no significance was observed in the total vascular bundle area in the stem between the two hybrids. Notably, XY335 had a 14.3 and 1.8% lower amount of root bleeding sap than that of ZD958 across the N0 and N150 levels, while 10.4% higher than that of ZD958 in the N300 level. Similar to the response of root bleeding-sap, treatments with N input showed dramatically higher MTE relative to those treatments without applied N. Additionally, greater MTE values were found for XY335 by 8.4% compared with those for ZD958 under N300 treatments (Table 5).

**TABLE 5:** Effect of nitrogen fertilization level on root bleeding sap and matter transport efficiency of basal-stem internode (MTE) of two maize hybrids at milking stage on N application levels in 2016.

Genotype	N level	Cross sectional area	vascular bundle area	Root bleeding sap	MTE
		(mm^2^)	(mm^2^)	(mg h^–1^)	(mg mm^–2^ h^–1^)
XY335	N0	242.7 ± 4.2e	25.8 ± 1.5c	537.5 ± 12.1e	20.0 ± 0.2f
	N150	275.7 ± 7.1d	32.3 ± 0.7b	979.2 ± 5.2c	30.4 ± 0.8d
	N300	365.3 ± 12.8b	56.5 ± 0.8a	2096.9 ± 10.3a	37.1 ± 0.4a
ZD958	N0	241.1 ± 8.0e	24.4 ± 0.5c	626.9 ± 23.5d	25.7 ± 0.9e
	N150	302.5 ± 8.6c	30.8 ± 0.9b	996.9 ± 4.6c	32.3 ± 0.8c
	N300	417.9 ± 14.8a	55.3 ± 2.1a	1878.1 ± 15.8b	34.0 ± 0.7b
ANOVA	Nitrogen (N)	***	***	***	***
	Genotype (G)	**	*	**	***
	N × G	*	NS	***	***

MTE, Matter transport efficiency. N0, N150, and N300 indicate 0, 150, and 300 kg ha^–1^ N applied, respectively.

Different small letters within a column indicate significant differences between treatments at a 5% level for each tissue position.

*, **, and *** indicate different significance at 5, 1, and 0.1% level, respectively; NS, no significance.

### Principal component analysis

**FIGURE 4:**
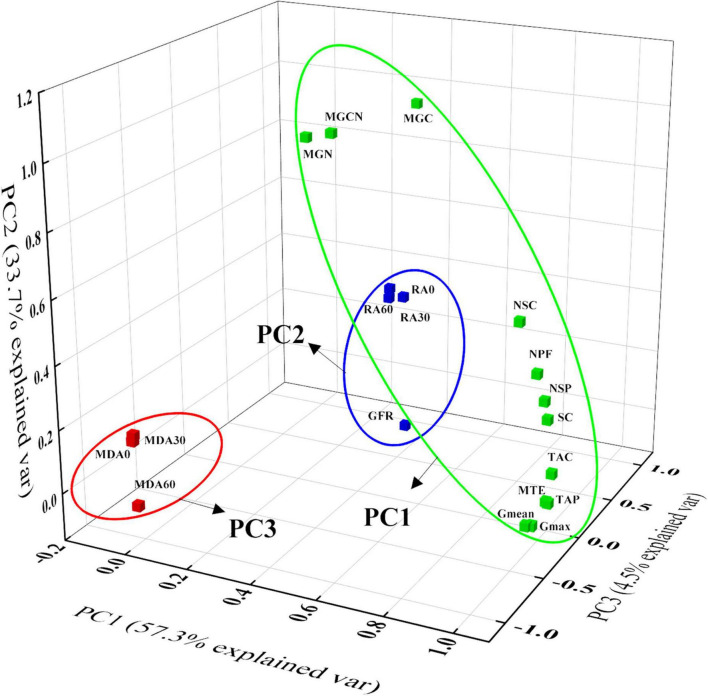
Principal component analysis (PCA) of grain filling parameters, vascular bundle structures, grain C, N contents, and leaf enzyme activity. *G*_*mean*_ and *G*_*max*_, mean, and maximum grain filling rate; MTE, matter transport efficiency; MGC, MGN, and MGCN, grain C, N contents, and C/N ratio at maturity stage; RA0, RA30, and RA60, root activity in 0–15 cm, 15–30 cm, and 30–60 cm soil layer at milking stage, respectively; MDA0, MDA30, MDA60, and malondialdehyde contents in 0–15 cm, 15–30 cm, and 30–60 cm soil layer at milking stage, respectively; NSP and NSC, number of small vascular bundle in peduncle and cob; TAP and TAC, total phloem area of small vascular bundle in peduncle and cob; GFP, active filling phase; NPF, number of pollinated florets; SC, Sink capacity.

Principal component analysis was employed to evaluate correlations between indicators tested in this study, and it showed that three principal components contributed to 57.3, 33.7, and 4.5% of the total variation. The 95.5% of the total variation in this study was explained by three principal components (Figure 4). Interestingly, we found that the grain-filling rates (*G*_*mean*_ and *G*_*max*_), total number of fertilized florets, matter transport efficiency of the basal-stem internode, grain C, N, and grain C/N ratio at the maturity stage, total phloem area of small vascular bundle in peduncle and cob tissue (TAC), and number of small vascular bundles in peduncle and cob tissues were more related to maize sink capacity than to other indicators included in PC1. Another cluster contained the root activity at the milking stage and the active-filling phase (GFR) was represented by PC2. In addition, root MDA contents at the milking stages contributed to PC3 (Figure 4).

## Discussion

### Nitrogen × Hybrids: Grain yield and sink capacity

Researchers have demonstrated that higher grain yield occurred through the superior sink capacity (KW × KNP) (31). An appropriate increased N application combined with right maize hybrids could improve dry matter accumulation and distribution to reproductive organs to achieve high sink capacity (15, 24, 25). In this study, the N rate, genotype, and their interaction affected maize sink capacity and grain yield. XY335 performed 9.3 and 9.8% lower sink capacity and grain yield than ZD958 under low N conditions (N0 and N150), whereas N × genotype interaction leads to higher sink capacity and grain yield in XY335 than ZD958 under N300 condition. Although XY335 had a lower 1,000 kernel weight (TKW) than that of ZD958, kernel number per ear (KNP) played a supportive role in compensating for the lower TKW. This compensation observed in XY335 likely contributes to its greater sink capacity and yield (Table 1). Furthermore, the higher KNP of XY335 was attributed to its greater fertilized florets, which was one of the key factors to determine the final kernel number (32, 33), and it is also influenced tremendously by N availability and crop genotype (34).

### Grain filling, sink capacity, and grain yield

Grain filling is an important indicator of sink potential and grain yield that significantly and positively correlate with photosynthetic assimilate production and translocation (4, 31, 35). Grain filling is driven by grain filling rate (GFR), grain filling period (GFP), or both, which were greatly affected by N application and genotype (2). Our previous research has shown that an appropriate increase in N application could achieve sufficient and efficient assimilates supply to grain, which contributed to the grain-filling rate for obtaining a higher grain yield of the same hybrid (9). However, our previous studies and other research mainly focused on the effect of grain filling on KW, and less information research on sink capacity in response to GFR or GFP between different genotype hybrids (32, 33, 35). Sink capacity is determined by KNP and potential kernel weight, and the maximum of single kernel weight is likely genetically determined, and thus the further supply of C assimilates could not raise the maximum weight (11, 13). In the determined model of increasing 100-kernel weight over time, we found that the grain weight of XY335 had reached 95.6% of its potential weight at approximately 45 days after pollination, while ZD958 had only reached 86.8% of its potential kernel weight at the same time (Figure 1), which resulted in a shorter GFP and higher *G*_*mean*_ and *G*_*max*_ for XY335 compared to those of ZD958 (Table 2). These results reveal that lower maximum single-kernel weight in XY335 contributed to relatively shorter GFP and higher KNP, which was likely the main factor influencing the increase in GFR. Results of the PCA analysis confirmed these results showing that the GFR belongs to PC1, while GFP is a part of PC2 (Figure 4). Previous studies also reported that GFR had a slightly and strongly positive correlation with GY than with GFP (4, 9, 35).

### Grain filling and C, N translocation and distribution

Simultaneously, the grain filling process of crops not only reflects assimilates supply, but also C and N transport and distribution between organs (36, 37). Increased N supply promotes larger quantities of carbohydrates translocating to grains, thereby increasing grain yield (38, 39). The present study demonstrated divergent responses in C and N contents in leaf tissues at the silking and maturity stages between two maize hybrids under conditions with various N supplies, which contributed to higher C and N contents in XY335 grains compared to the corresponding contents in ZD958 grains under N300 conditions. In addition, according to the ^13^C tracer analysis at maturity, generally lower ^13^C assimilates in XY335 were distributed in the stem, leaf (ear leaf and other leaf), and sheath tissues, but higher values were distributed in husk leaf, grain, and cob tissues compared to those in ZD958, especially in N300 conditions (Table 3). These results reflect the higher MTE from source to sink in XY335 relative to that in ZD958, which was likely attributed to the higher GFR in XY335. Furthermore, the C/N ratio plays a greater role in matter translocation between crop tissues rather than C or N contents individually, balance C/N ratio within crops can regulate assimilates translocation from leaves to grains, thereby increasing dry matter accumulation and grain matter (36, 40). In our case, a lower C/N ratio was observed in XY335 grains than that in ZD958 grains at the maturity stage of from N0 and N150 groups, while there were higher ratios of XY335 at the treatment of N300. Also, higher C and N contents were measured in XY335 grains than in ZD958 grains as mentioned above (Figure 2). Thus, it could be concluded that not only a stronger C and N translocation from the vegetative organs to grains, but also balanced C/N ratios are required in maize grains applied with appropriate N levels, which is also important in regulating the grain-filling process to achieve high grain yield.

### Grain filling is associated with the bleeding sap and vascular bundle structure

Research on bleeding sap primarily aimed to elucidate the mechanism of matter transfer from roots to shoots (41). Bleeding-sap transport nutrient matter between aboveground and underground, which represents the higher amount of N and kernel number, may explain the variations of grain filling between two maize genotypes supplied with contrasting N fertilizer (42, 43). The collected bleeding sap indicated that a lower bleeding sap ratio was observed in XY335 than that in ZD958 at the milking stage in N0 and N150 groups, while there were significantly higher values in XY335 than that from ZD958 at N300 treatment (10.4%). In this study, similar results were also observed for root activity (Figure 3). Morita et al. (44) and Noguchi et al. (45) reported that the root-bleeding rate was closely related to root traits in maize, and it could be used to evaluate the physiological activity of root activity. Strong root activity is necessary to increase the accumulation of post-silking dry matter and grain filling (46, 47). Moreover, higher MDA contents will enhance superoxide enzyme activity, which leads to plant senescent (48). XY335 exhibited lower MDA contents than ZD958 (Figure 3). These findings suggested that the N rate significantly increased root activity and decreased MDA content, thus boosting higher bleeding sap in XY335 than that in ZD958 under sufficiency N application.

The structure of the vascular bundle, as the main channel, determines bleeding sap transport ability (42, 49). They also regulate endosperm C metabolites through translocating sugars and N between tissues (13). For instance, as much as 80% of C assimilated in leaves was transported *via* the phloem to satisfy the metabolic needs of other plant organs (50). Increasing N application can increase the number of small vascular bundles to boost kernel number, and enhanced phloem areas of small vascular bundles are beneficial for assimilation transport to grain (9), determining the total accumulation of assimilation in sink capacity, which affects grain filling characteristic under various conditions (9, 51, 52). However, how the N × genotype interaction changes the number and area of the vascular bundle, and the relationship between the vascular bundle characteristic and the grain filling are still unclear. In this study, there was no significant difference in NSP and NSC under lower N conditions between the two hybrids, while the N300 treatment significantly increased both NSP and NSC more in XY335 than those in ZD958 (Table 4). Moreover, the TAP and TAC values showed similar trends to those of NSP and NSC as N inputs increased (Table 4). Higher NSP and NSC together with larger TAP and TAC contributed to the significantly higher MTE in XY335 relative to that of ZD958 (Table 5). The PCA analysis showed that TAC, NSP, NSC, and MTE correlated well with PC1 (Figure 4), suggesting that these responses related to vascular bundles in XY335 are especially important in promoting bleeding sap and grain filling. The better vascular system benefited MTE, and simultaneously might contribute to the increase in GFR and C and N translocation to florets, which ultimately resulted in the final stronger sink capacity and grain yield (53).

## Conclusion

**FIGURE 5:**
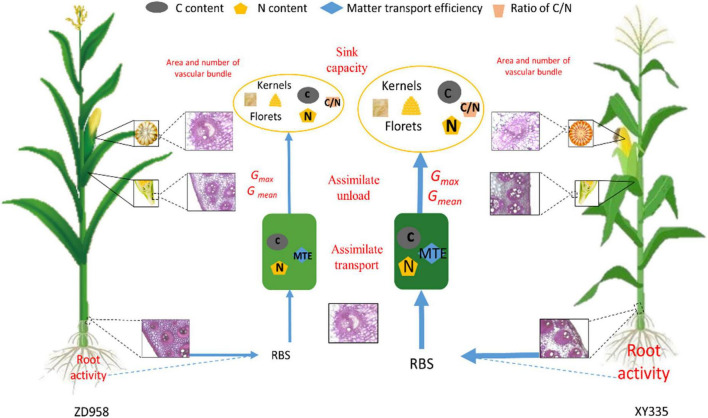
Schematic from this study showing the differences in filling parameters and the response of vascular bundle structure, matter transportation, and source–sink relationship on N input levels between maize varieties ZD958 and XY335. RBS, root bleeding sap; MTE, matter transport efficiency; *G*_*mean*_ and *G*_*max*_, mean and maximum grain filling rate; GY, grain yield. Different sizes of the same letter represent value differences.

The factors of crop genotype and N fertilizer interacted with optimization of vascular bundle structure of ear tissue in XY335, thus increasing 10.4% bleeding sap and 8.4% MTE than those in ZD958 under N300 condition. Moreover, the regulation of the C/N ratio in XY335 under higher levels of N treatments provided more C assimilates to facilitate floret development and increase the final kernel number. Therefore, these results indicate that the sufficient N input can improve root activity and optimize the vascular bundle system in the ear to boost matter transport efficiency, in turn, increase the transport of C and N into grains and balance the C/N ratio in XY335, which promote a favorable grain filling rate ultimately for enhancing sink capacity and grain yield (Figure 5). These findings, to some extent, could be used to inform maize breeding and cultivation that higher grain-filling rate, sink capacity, and allocation of matter into kernels are significant factors for striving to attain higher grain yields. Moreover, future studies should also focus on optimizing the vascular bundle system in maize peduncle and cob tissues to improve grain yields.

## Data availability statement

The original contributions presented in this study are included in the article/supplementary material, further inquiries can be directed to the corresponding authors.

## Author contributions

HR: methodology, investigation, data curation, writing—original draft, and funding acquisition. MZ and HQ: formal analysis and resources. BZ: investigation, methodology, and editing. WZ: methodology and data curation. KL: formal analysis. YJ: conceptualization, methodology, writing—review and editing, supervision, and project administration. CL: conceptualization, methodology, resources, writing—review and editing, supervision, project administration, and funding acquisition. All authors read and approved the article.

## References

[1] Kato T Takeda K. Associations among characters related to yield sink capacity in space−planted rice. *Crop Sci.* (1996) 36:1135–9. 10.2135/cropsci1996.0011183X003600050011x

[2] Zhou BY Yue Y Sun XF Ding ZS Ma W Zhao M. Maize kernel weight responses to sowing date-associated variation in weather conditions. *Crop J.* (2017) 5:43–51. 10.1016/j.cj.2016.07.002

[3] Uribelarrea M Below FE Moose SP. Grain composition and productivity of maize hybrids derived from the Illinois protein strains in response to variable nitrogen supply. *Crop Sci.* (2004) 44:1593–600. 10.2135/cropsci2004.1593

[4] Wei SS Wang XY Li GH Qin YY Jiang D Dong ST. Plant density and nitrogen supply affect the grain-filling parameters of maize kernels located in different ear positions. *Front Plant Sci.* (2019) 10:180. 10.3389/fpls.2019.00180 30881365 PMC6405445

[5] Arisnabarreta S Miralles DJ. Radiation effects on potential number of grains per spike and biomass partitioning in two- and six-rowed near isogenic barley lines. *Field Crops Res.* (2008) 107:203–10. 10.1016/j.fcr.2008.01.005

[6] Sandana PA Harcha CI Calderini DF. Sensitivity of yield and grain nitrogen concentration of wheat, lupin and pea to source reduction during grain filling. a comparative survey under high yielding conditions. *Field Crops Res.* (2009) 114:233–43. 10.1016/j.fcr.2009.08.003

[7] Ishibashi Y Okamura K Miyazaki M Phan T Yuasa T Iwaya-Inoue M. Expression of rice sucrose transporter gene OsSUT1 in sink and source organs shaded during grain filling may affect grain yield and quality. *Environ Exp Bot.* (2014) 97:49–54. 10.1016/j.envexpbot.2013.08.005

[8] Liu GZ Yang YS Guo XX Liu WM Xie RZ Ming B Coordinating maize source and sink relationship to achieve yield potential of 22.5 Mg ha^–1^. *Field Crop Res.* (2022) 283:108544. 10.1016/j.fcr.2022.108544

[9] Ren H Jiang Y Zhao M Qi H Li CF. Nitrogen supply regulates vascular bundle structure and matter transport characteristics of spring maize under high plant density. *Front Plant Sci.* (2020) 8:602739. 10.3389/fpls.2020.602739 33488648 PMC7820718

[10] Cárcova J Uribelarrea M Borrás L Otegui ME Westgate ME. Synchronous pollination within and between ears improves kernel set in maize. *Crop Sci.* (2000) 40:1056–61. 10.2135/cropsci2000.4041056x

[11] Borrás L Slafer GA Otegui ME. Seed dry weight response to source-sink manipulations in wheat, maize, and soybean: a quantitative reappraisal. *Field Crop Res.* (2004) 86:131–46. 10.1016/j.fcr.2003.08.002

[12] Paponov IA Sambo P Erley G Presterl T Geiger HH Engels C. Kernel set in maize genotypes differing in nitrogen use efficiency in response to resource availability around flowering. *Plant Soil.* (2005) 272:101–10. 10.1007/s11104-004-4210-8

[13] Cázetta JO Seebauer JR Below FE. Sucrose and nitrogen supplies regulate growth of maize kernels. *Ann Bot-London.* (1999) 84:747–54. 10.1006/anbo.1999.0976

[14] Andersen MN Asch F Wu Y Jensen CR Naested H Mogensen VO Soluble invertase expression is an early target of drought stress during the critical, abortion sensitive phase of young ovary development in maize. *Plant Physiol.* (2002) 130:591–604. 10.1104/pp.005637 12376627 PMC166589

[15] D’Andrea KE Otegui ME Cirilo AG. Kernel number determination differs among maize hybrids in response to nitrogen. *Field Crop Res.* (2008) 105:228–39. 10.1016/j.fcr.2007.10.007

[16] Kiniry JR Tischler CR Rosenthal WD Gerik TJ. Nonstructural carbohydrate utilization by sorghum and maize shaded during grain growth. *Crop Sci.* (1992) 32:131–7. 10.2135/cropsci1992.0011183X003200010029x

[17] Zhu GH Ye NH Yang JC Peng XX Zhang J. Regulation of expression of starch synthesis genes by ethylene and ABA in relation to the development of rice inferior and superior spikelets. *J Exp Bot.* (2011) 62:3907–16. 10.1093/jxb/err088 21459770 PMC3134347

[18] D’Andrea KE Piedra CV Mandolino CI Cirilo AG Otegui ME. Contribution of reserves to kernel weight and grain yield determination in maize: phenotypic and genotypic variation. *Crop Sci.* (2016) 56:697–706. 10.2135/cropsci2015.05.0295

[19] Li YB Tao HB Zhang BC Huang SB Wang P. Timing of water deficit limits maize kernel setting in association with changes in the source-flow-sink relationship. *Front. Plant Sci.* (2018) 9:1326. 10.3389/fpls.2018.01326 30405644 PMC6204571

[20] Bialczyk J Lechowski Z. Chemical composition of xylem sap of tomato grown on bicarbonate containing medium. *J Plant Nutr.* (1995) 18:2005–21. 10.1080/01904169509365040

[21] Peuke AD. The chemical composition of xylem sap in vitis vinifera L.cv, Riesling during vegetative vineyard soils and as influenced by nitrogen fertilizer. *Am J Enol Viticult.* (2000) 51:329–39.

[22] Lalonde S Tegeder M Throne−Holst M Frommer WB Patrick JW. Phloem loading and unloading of sugars and amino acids. *Plant Cell Environ.* (2003) 26:37–56. 10.1046/j.1365-3040.2003.00847.x

[23] Feng HJ Zhang SP Ma CJ Liu P Dong ST Zhao B Effect of plant density on microstructure of stalk vascular bundle of summer maize (*Zea mays* L.) and its characteristics of sap flow. *Acta Agron Sin.* (2014) 40:1435–42. 10.3724/SP.J.1006.2014.01435

[24] Ciampitti IA Vyn TJ. Physiological perspectives of changes over time in maize yield dependency on nitrogen uptake and associated nitrogen efficiencies: a review. *Field Crop Res.* (2012) 133:48–67. j.fcr.2012.03.008 10.1016/j.fcr.2012.03.008

[25] Peng Y Li C Fritschi FB. Apoplastic infusion of sucrose into stem internodes during female flowering does not increase grain yield in maize plants grown under nitrogen−limiting conditions. *Physiol Plantarum.* (2013) 148:470–80. 10.1111/j.1399-3054.2012.01711.x 23061679

[26] Duncan DR Widholm JM. Osmotic induced stimulation of the reduction of the viability dye 2,3,5-triphenyltetrazolium chloride by maize roots and callus cultures. *J Plant Physiol.* (2004) 161:397–403. 10.1078/0176-1617-01237 15128027

[27] Liu T Gu L Dong S Zhang J Liu P Zhao B. Optimum leaf removal increases canopy apparent photosynthesis, 13C-photosynthate distribution and grain yield of maize crops grown at high density. *Field Crop Res.* (2015) 170:32–9. 10.1016/j.fcr.2014.09.015

[28] Piao L Qi H Li C Zhao M. Optimized tillage practices and row spacing to improve grain yield and matter transport efficiency in intensive spring maize. *Field Crop Res.* (2016) 198:258–68. 10.1016/j.fcr.2016.08.012

[29] Liu X Wang X Wang X Gao J Luo N Meng Q Dissecting the critical stage in the response of maize kernel set to individual and combined drought and heat stress around flowering. *Environ Exp Bot.* (2020) 179:104213. 10.1016/j.envexpbot.2020.104213

[30] Yoshinaga S Takai T Arai-Sanoh Y Ishimaru T Kondo M. Varietal differences in sink production and grain-filling ability in recently developed high yielding rice (Oryza sativa L.) varieties in Japan. *Field Crops Res.* (2013) 150:74–82. 10.1016/j.fcr.2013.06.004

[31] Yagioka A Satoshi H Kenji KM. Sink production and grain-filling ability of a new high-yielding rice variety, Kitagenki. *Field Crop Res.* (2021) 260:107991. 10.1016/j.fcr.2020.107991

[32] Gonzalez VH Lee EA Lukens LN Swanton CJ. The relationship between floret number and plant dry matter accumulation varies with early season stress in maize (*Zea mays* L.). *Field Crop Res.* (2019) 238:129–38. 10.1016/j.fcr.2019.05.003

[33] Zhu YG Chu JP Dai XL He MR. Delayed sowing increases grain number by enhancing spike competition capacity for assimilates in winter wheat. *Eur J Agron.* (2019) 104:49–62. 10.1016/j.eja.2019.01.006

[34] Parco M Ciampitti IA D’Andrea KE Maddonni G. Prolificacy and nitrogen internal ef–ficiency in maize crops. *Field Crops Res.* (2020) 256:107–12. 10.1016/j.fcr.2020.107912

[35] Ren H Qi H Zhao M Zhou WB Wang XB Gong XW Characterization of source–sink traits and carbon translocation in maize hybrids under high plant density. *Agron J.* (2022) 12:961. 10.3390/agronomy12040961

[36] Pommel B Gallais A Coque M Quillere I Hirel B Prioul JL Carbon and nitrogen allocation and grain filling in three maize hybrids differing in leaf senescence. *Eur J Agron.* (2006) 24:203–11. 10.1016/j.eja.2005.10.001

[37] Liu K Ma BL Luan L Li C. Nitrogen, phosphorus, and potassium nutrient effects on grain filling and yield of high-yielding summer corn. *J Plant Nutr.* (2011) 34:1516–31. 10.1080/01904167.2011.585208

[38] Zhang H Han K Gu S Wang D. Effects of supplemental irrigation on the accumulation, distribution and transportation of 13C-photosynthate, yield and water use efficiency of winter wheat. *Agr Water Manage.* (2019) 214:1–8. 10.1016/j.agwat.2018.12.028

[39] Paul MJ Driscoll SP. Sugar repression of photosynthesis: the role of carbohydrates in signalling nitrogen deficiency through Source: sink imbalance. *Plant Cell Environ.* (1997) 20:110–6. 10.1046/j.1365-3040.1997.d01-17.x

[40] Dong H Li W Eneji A Zhang D. Nitrogen rate and plant density effects on yield and late–season leaf senescence of cotton raised on a saline field. *Field Crop Res.* (2012) 126:137–44. 10.1016/j.fcr.2011.10.005

[41] Engels C Marschner H. Influence of the form of nitrogen supply on root uptake and translocation of cations in the xylem exudate of maize (*Zea mays* L.). *Environ. Exp. Bot.* (1993) 44:1695–701. 10.1093/jxb/44.11.1695 12432039

[42] Fukuyama T Takayama T. Variations of the vascular bundle system in Asian rice cultivars. *Euphytica.* (1995) 86:227–31. 10.1007/BF00016360

[43] Zhang ZH Li P Wang LX. Identification of quantitative trait loci (QTLs) for the characters of vascular bundles in peduncle related to indica-japonica differentiation in rice (*Oryza sativa* L.). *Euphytica.* (2002) 128:279–84. 10.1023/A:1020802001207 12645263

[44] Morita S Okamoto M Abe J Yamagishi J. Bleeding rate of field-grown maize with reference to root system development. *Jpn J Crop Sci.* (2000) 69:80–5. 10.1626/jcs.69.80

[45] Noguchi A Kageyama M Shinmachi F. Potential for using plant xylem sap to evaluate inorganic nutrient availability in soil: i. influence of inorganic nutrients present in the rhizosphere on those in the xylem sap of luffa cylindrica roem.(soil fertility). *Soil Sci Plant Nutr.* (2005) 51:333–41. 10.1111/j.1747-0765.2005.tb00038.x

[46] Li H Liu L Wang Z Yang J Zhang J. Agronomic and physiological performance of high-yielding wheat and rice in the lower reaches of Yangtze River of China. *Field Crop Res.* (2012) 133:119–29. 10.1016/j.fcr.2012.04.005

[47] Guan D Al-Kaisi MM Zhang Y Duan L Tan W Zhang M Tillage practices affect biomass and grain yield through regulating root growth, root-bleeding sap and nutrients uptake in summer maize. *Field Crop Res.* (2014) 157:89–97. 10.1016/j.fcr.2013.12.015

[48] Miao BH Han XG Zhang WH. The ameliorative effect of silicon on soybean seedlings grown in potassium-deficient medium. *Ann Bot-London.* (2010) 105:967–73. 10.1093/aob/mcq063 20338952 PMC2876006

[49] He QP Dong ST Gong RQ. Comparison of ear vascular bundles in different maize cultivars. *Acta Agron Sin.* (2010) 33:1187–96.

[50] Kalttorres W Kerr PS Usuda H Huber SC. Diurnal changes in maize leaf photosynthesis: i. Carbon exchange rate, assimilate export rate, and enzyme activities. *Plant Physiol.* (1987) 83:283–8. 10.1104/pp.83.2.283 16665237 PMC1056349

[51] Yang JC Zhang JH Wang ZQ Zhu QS Liu LJ. Water deficit–induced senescence and its relationship to the remobilization of prestored carbon in wheat during grain filling. *Agron J.* (2001) 93:196–206. 10.2134/agronj2001.931196x

[52] Monneveux P Zaidi PH Sanchez C. Population density and low nitrogen affects yield associated traits in tropical maize. *Crop Sci.* (2005) 45:535–45. 10.2135/cropsci2005.0535

[53] Huang X Qian Q Liu Z Sun H He S Luo D Natural variation at the DEP1 locus enhances grain yield in rice. *Nat Genet.* (2009) 41:494–7. 10.1038/ng.352 19305410

